# Simulation-based development: shaping clinical procedures for extra-uterine life support technology

**DOI:** 10.1186/s41077-023-00267-y

**Published:** 2023-12-02

**Authors:** J. S. van Haren, M. B. van der Hout-van der Jagt, N. Meijer, M. Monincx, F. L. M. Delbressine, X. L. G. Griffith, S. G. Oei

**Affiliations:** 1 Department of Industrial Design, Eindhoven University of Technology, Eindhoven, The Netherlands; 2 Department of Obstetrics & Gynecology, Máxima Medisch Centrum, Veldhoven, The Netherlands; 3 Department of Electrical Engineering, Eindhoven University of Technology, Eindhoven, The Netherlands; 4 Department of Biomedical Engineering, Eindhoven University of Technology, Eindhoven, The Netherlands

**Keywords:** Simulation-based development, Co-creation, Participatory design, Perinatal life support technology, Artificial placenta, Artificial womb, Extra-uterine life support

## Abstract

**Background:**

Research into Artificial Placenta and Artificial Womb (APAW) technology for extremely premature infants (born < 28 weeks of gestation) is currently being conducted in animal studies and shows promising results. Because of the unprecedented nature of a potential treatment and the high-risk and low incidence of occurrence, translation to the human condition is a complex task. Consequently, the obstetric procedure, the act of transferring the infant from the pregnant woman to the APAW system, has not yet been established for human patients. The use of simulation-based user-centered development allows for a safe environment in which protocols and devices can be conceptualized and tested. Our aim is to use participatory design principles in a simulation context, to gain and integrate the user perspectives in the early design phase of a protocol for this novel procedure.

**Methods:**

Simulation protocols and prototypes were developed using an iterative participatory design approach; usability testing, including general and task-specific feedback, was obtained from participants with clinical expertise from a range of disciplines. The procedure made use of fetal and maternal manikins and included animations and protocol task cards.

**Results:**

Physical simulation with the active participation of clinicians led to the diffusion of tacit knowledge and an iteratively formed shared understanding of the requirements and values that needed to be implemented in the procedure. At each sequel, participant input was translated into simulation protocols and design adjustments.

**Conclusion:**

This work demonstrates that simulation-based participatory design can aid in shaping the future of clinical procedure and product development and rehearsing future implementation with healthcare professionals.

**Supplementary Information:**

The online version contains supplementary material available at 10.1186/s41077-023-00267-y.

## Background

Extremely preterm infants, born before 28 weeks of gestation, often present with pathologies that could include respiratory distress syndrome, cerebral hemorrhage, sepsis, retinopathy, and/or developmental deficits [1]. To support growth and development, newborns are placed in warmed incubators with respiratory support [2]. The fetal and neonatal scientific community continues to research a range of treatments for extreme preterm birth to ensure better outcomes for these infants. Recently, Artificial Placenta (AP) and Artificial Womb (AW), or (APAW) technology are being studied as an alternative [3]. APAW is an extra-uterine life support system that simulates the liquid environment of the native womb while also supplying oxygen to the perinate [4]. Multiple animal experiments have successfully maintained fetal lambs for up to 28 days on artificial placenta support [5], bringing the technology closer to clinical application [6]. The innovative cardiovascular intervention, oxygenators, umbilical cord cannulation, and liquid environment design to prevent sepsis were the focus of these studies. For a patient, the first step of such a treatment would entail the infant’s birth and the subsequent transfer from the native womb into the artificial womb. In previous animal studies, this entailed a cesarean section (CS) which typically was performed under general anesthesia, thereby consequently suppressing the breathing reflex. Lung aeration may trigger the fetal to neonatal cardiovascular transition [7] and would make the use of APAW unsuitable as the aim is to supply oxygen-rich blood via the umbilical cord, not the lungs, to maintain a fetal physiological state. An investigation into the obstetric procedure has not been described thus far. Such an unprecedented CS and vaginal birth (VB) procedure demands new protocols, new collaborations, adaptability to the new socio-technical environment, and perhaps even new disciplines to join the operating room. In this study, we employ a simulation-based development method to form an understanding of the considerations related to various obstetric practices. Through this methodology, we gain and integrate perspectives from different stakeholders in the early design phase of suitable obstetric protocols for a simulated transfer by CS and VB.

### Simulation

Animal models have helped advance scientific fields including obstetrics [8] and premature birth [9]. Much of our knowledge regarding APAW technology stems from studies conducted on animals, contributing predominantly and being essential to the advancement of this field. Pre-clinical studies have been used to evaluate APAW systems [5, 10, 11], but further investigation into certain topics is appropriate before moving to human translation with a fully developed system [4, 12]. Animal research and laboratory data may not match clinical outcomes. This translation adds new challenges and requirements that need to be identified and addressed. These include the careful consideration of ethical issues for human studies [12, 13]. The unpredictable nature of premature births, the critical high-risk therapeutic uncertainties, the emotional load on patients’ families and clinical staff, and low patient recruitment for a first-in-human study, make regular clinical studies difficult to achieve [14].

To approximate the human patient and allow for improvement of the procedure, protocol, and system before performing human trials, one can choose from a range of methods including using human tissue, synthetic tissue, human cadavers, inanimate models, interactive human patient simulators and virtual/augmented reality [15, 16]. A promising alternative to successfully develop life-support technologies, distill user insights, and improve quality of care can be found in the creation of a high-fidelity medical simulation. This method has already proven to be a successful educational tool for medical training [17–20]. Simulation can allow the testing of high-risk but low-frequency clinical scenarios and thereby optimize APAW systems for a wider range of pathologies.

### Simulation-based user-centered design

With simulation, research on medical safety and technical feasibility could also be accompanied by ongoing research on usability and socio-ethical aspects. To form an understanding of the requirements of a transfer procedure for human patients and assess clinical acceptance, APAW research should collaborate with various stakeholders. These include parents, medical experts (such as nurses, obstetricians, perinatologists, fetal surgeons, and neonatologists), medical engineers, and potentially new specialists essential for performing this treatment. Frequent stakeholder involvement may improve safety (including human error reduction), effectiveness, and user satisfaction [21–23]. Clinical team dynamics can be analyzed, workflows can be established, and crucial communication and decision moments can be structured in a clinical protocol. Tacit knowledge insights from clinicians are crucial throughout obstetric procedure development.

Various terminologies exist to define the collaboration between academics and multiple stakeholders in the design process, including co-creation [24], co-design [25], participatory design [26], co-production [27] and clinician-driven design [28]. These terms are often used interchangeably, all moving away from the concept of a design *for* users, to a design *by* users. Through the involvement of stakeholders in the design process [24–28], it is ensured that the resulting outcome meets all needs and expectations*.*

This study proposes participatory simulation to elicit environment-specific user insights for APAW protocol, procedure, and device development. Although protocol, procedure, and device development all progressed in parallel, in this study we focus on the development of a simulation protocol that provides a step-by-step plan for the transfer of a fetus from the maternal uterus to the APAW system. The study examined anesthetic management, uterine relaxation, fetal environmental exposure, maternal positioning, amniotic fluid management, vital function monitoring, hygiene, and mode of transfer.

## Methods

### Protocol template

To formulate procedure requirements, fetal physiology insights were gained from literature and prior APAW studies. Initial protocols stemmed from established medical guidelines, such as those by the Dutch Society for Obstetrics and Gynecology (NVOG, the Netherlands) and Máxima Medical Center (the Netherlands). The study population focuses on extremely premature infants (24–28 weeks gestation), constituting 5% of preterm births globally [29]. Eligibility criteria, based on preterm labor guidelines [30, 31], prevent undue complexity during this early phase of research. Therefore, the vaginal delivery transfer excludes scenarios such as placenta previa, breech position, and multiple pregnancies. Cesarean transfer omits abruptio placentae and multiple pregnancies, with potential changes during further development.

### Simulation setup

**Fig. 1:**
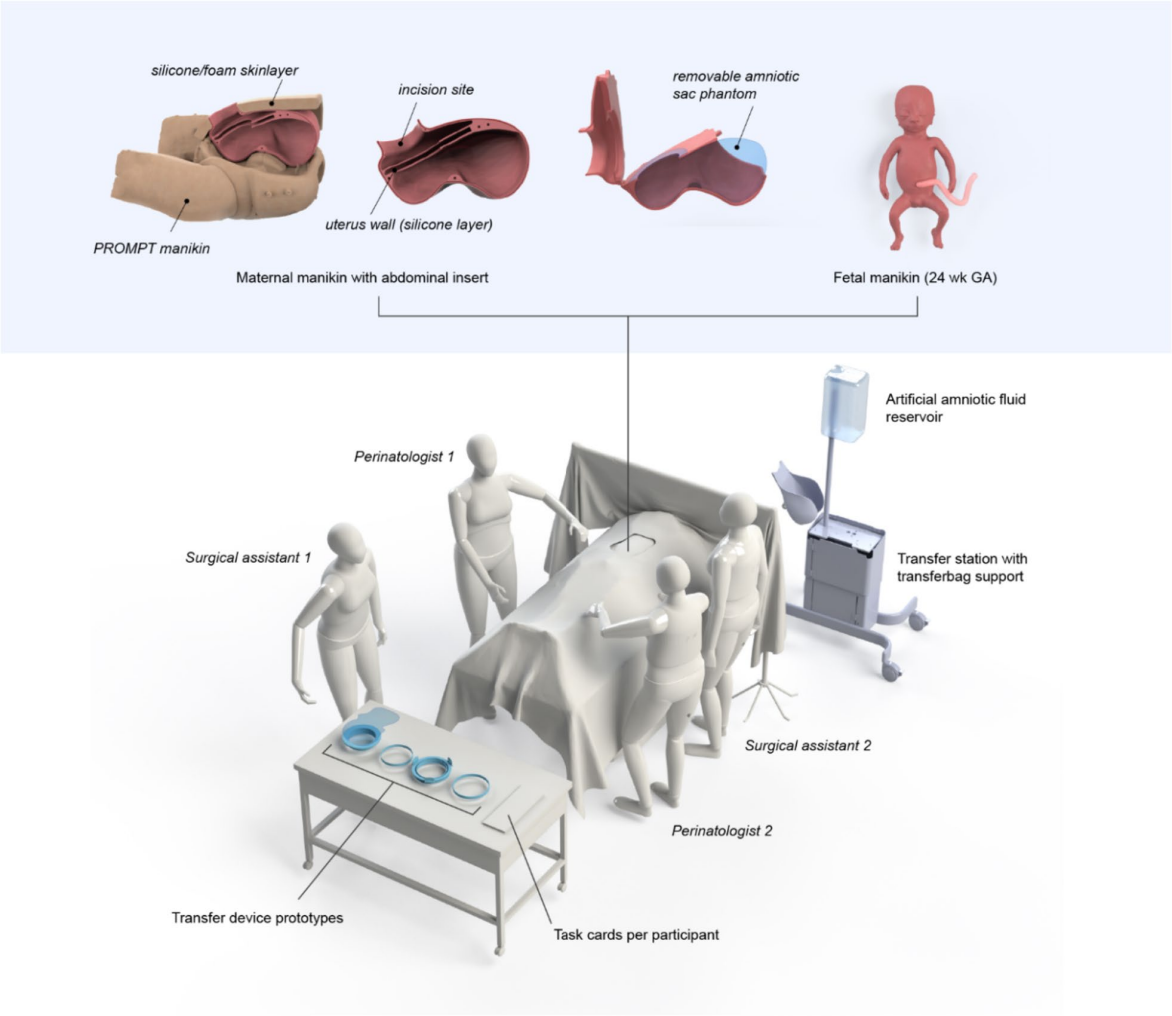
Setup of the physical simulation room in phase V

MRI data of a 24-week fetus were used to create a custom silicone manikin. A PROMPT Flex Birthing Simulator (Limbs and Things, United Kingdom) simulated the mother’s abdomen. The manikin was 3D scanned (Eva Scanner, Artec 3D, Luxembourg) and 3D printed (PLA filament, Ultimaker, the Netherlands) to create an abdominal insert (see Fig. 1). Transfer devices aiding the procedure included a transparent bag with integrated gloves and a stiff outer ring (transferbag) that can be attached to the incision site using wound retractors [32]. The ensemble of tools is referred to as the transfer device and acts as a discussion starter rather than a finalized instrument.

### Participant recruitment

To distill existing preterm care knowledge and generate new insights for the novel procedure, we employed semi-structured interviews and medical simulations. Participants, chosen for their clinical expertise in preterm delivery and care, were recruited via research consortium connections and snowball sampling. Phase I interviews occurred through videoconferencing; subsequent phases were in-person or by videoconferencing. The medical simulations were all conducted in-person.

**Fig. 2:**
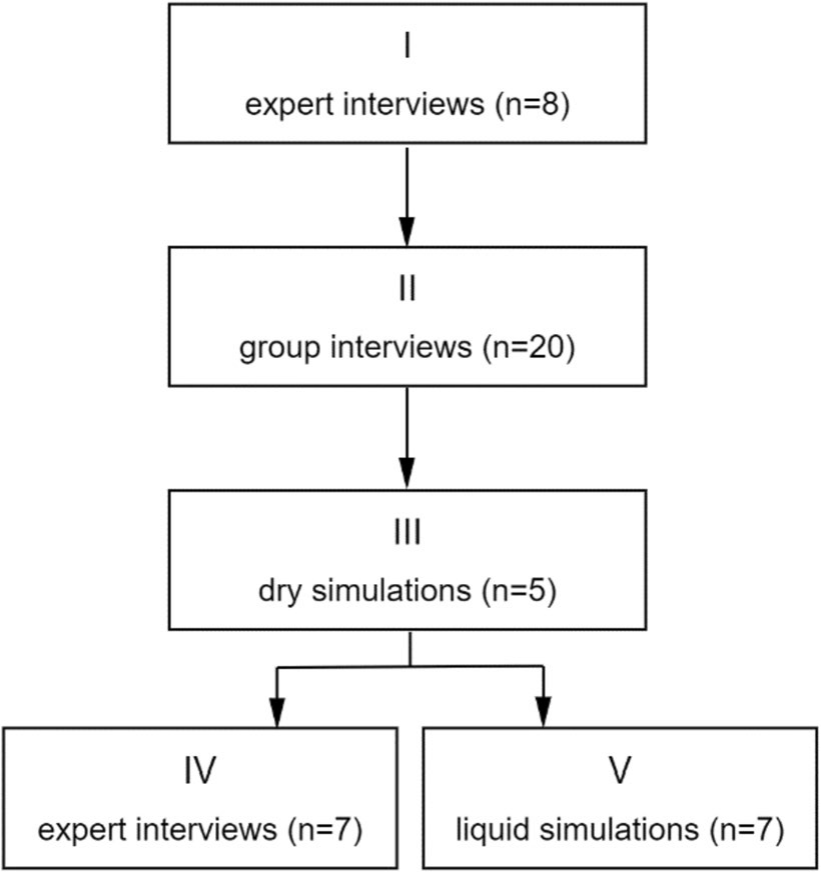
Participant and phase overview

Participant expertise increased as iterations progressed toward a functional protocol. Medical residents contributed to concept development, while senior professionals offered targeted insights. Especially at the start of development, certain aspects required more attention. Individual simulation sessions focused on the specialization of the participant while the researcher acted out other tasks necessary to perform a full procedure. Interruptions to clinical practice were minimized by dosing the time investment needed. Participant involvement is summarized in Fig. 2.

### Grounded theory

To build a common understanding regarding the procedure requirements, methods from a grounded theory approach were employed [33]. This qualitative method involves analyzing existing and newly acquired data from interviews and simulations. Grounded theory's value lies in its iterative process: data gathering (literature, interviews, simulations), initial analysis, continued sampling, further analysis, idea testing, and concept refinement, iterating until saturation is reached and novel perspectives cease to emerge [34].

### Phases

The protocol development consisted of five phases. The initial phase included exploratory expert interviews with eight medical professionals from the Obstetrics and Gynecology department at Máxima Medical Center (the Netherlands)—comprising two obstetricians, five physicians, and a neonatology intensive care nurse (P1–P8). After an initial draft, 20 participants engaged in six expert interviews (P9–P28), where a slideshow introduced the protocol concept. Focus lay on discussing challenges and possible alternatives, supplemented by relevant literature. Tailored inquiries probed experts’ knowledge for detailed insights. Due to the novelty of the procedure, visual aids such as renderings, illustrations, or animations were utilized for clarity.

Dry simulations were held with five participants (P29–P35), including three perinatologists, a technical physician, and a medical engineer with midwifery experience. Sessions took place at Eindhoven University of Technology (the Netherlands) and Medsim (the Netherlands), utilizing fetal and maternal manikins. Participants were instructed on procedural tasks, tool handling, and their place within the entire clinical team. Only tasks related to non-pharmacological treatments were included. Prototype-assisted discussions and idea generation occurred, supplemented by illustrations, animations, or live instructions. Task cards aided task sequencing. Phase IV included seven expert interviews with diverse disciplines - three perinatologists, two anesthesiologists, and two neonatologists (P36–P42). The semi-structured interview guide drew from hospital protocols, literature on preterm infant care, and the current transfer procedure concept [35]. Additional file 1 contains the interview questions.

**Fig. 3:**
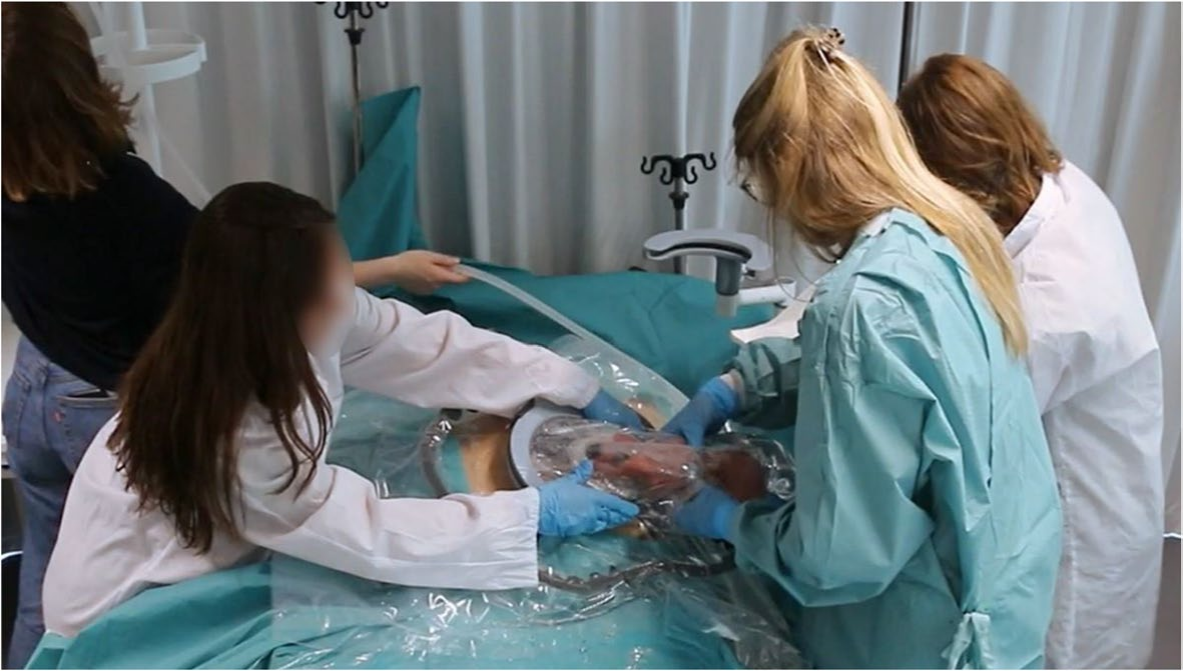
Simulation session of a liquid-based transfer procedure with the manikin being delivered into a transferbag

Liquid-based simulations were conducted with seven participants (P43–P49) at Eindhoven University of Technology. Figures 1 and 3 display the manikins and tools used. The simulation of pharmacological treatments was omitted. The transfer simulations included in this study were only for liquid-based CS, not for vaginal birth. Expert interviews ranged from 29 to 98 min, while each single simulation (excluding preparation and debrief) lasted about 10–15 min, with participants taking on each role once.

### Data analysis

Transcripts were made from the recordings, when recording was not possible minutes were made directly after the session. Continuous comparison of interview and simulation insights against prior information ensured internal validity. Through data and source triangulation, internal validity was assured. Data triangulation was assured using a variety of methods such as simulation and individual/group interviews. Source triangulation was assured by including specialists from different disciplines and sources of experience.

## Results

The knowledge generated in this study is two-fold. Initial insights stem from expert interviews and simulations, addressing clinical and user aspects. Second, an evolving protocol emerged, refining iteratively after each session.

### Insights

Sampling, data collection, and analysis were interleaved processes. The grounded theory approach gathered literature study, expert input, and simulation findings to construct a shared understanding of needs. The synthesized insights, with illustrative quotes, are grouped into different themes, namely, mode of transfer, anesthesia, uterine relaxants, fetal environmental exposure, maternal positioning, artificial amniotic fluid (AAF) flow into the uterus, arterial line, monitoring of fetal vitals, fetal vascular access, and hygiene.

### Mode of transfer

During expert discussions (P26–P28), concerns arose regarding the feasibility of an effective transfer after vaginal delivery. Participants (P21, P22, P26–P28) recognized that irreversible physiological shifts could occur upon labor or passage through the vaginal canal, prompting fetal transition to neonatal physiology. To enhance transfer success and considering the prominence of cesarean sections in preterm births [36–39], our study expanded to also formulate a cesarean transfer procedure. Participants (P26–P28) believed that a cesarean approach offered advantages such as more time to perform the transfer, controlled conditions, improved fetal monitoring, and reduced risk of unintended physiological transition.

### Anesthesia and pharmacological treatment

Previous animal APAW studies administered drugs to premedicate, anesthetize, intubate, and ventilate ewes [5, 11]. General anesthesia led to fetal lamb respiratory suppression, particularly through buprenorphine and propofol, which are known for their ventilation-depressing properties [40]. These opioids and anesthetics (commonly propofol and sevoflurane during CS in the Netherlands) cross the placenta [41–43]. General anesthesia is typically avoided in standard cesarean sections [44] and is used only during emergencies or medical necessity (e.g., umbilical cord prolapse, low platelets) [45] [46–48]. During human fetal surgeries, such as ex-utero intrapartum treatment, general anesthesia is also used [41, 49, 50].

Despite this, 6 of 7 participants in phase IV (P36–P41) favored general anesthesia over spinal anesthesia, citing benefits such as fetal respiration suppression, uterine relaxation, and reduced time pressure to perform the transfer. A drawback was mentioned (P42)—the potential negative impact on maternal mental well-being, affecting the conscious delivery experience and parent-infant bonding. “I think especially from a psychological point of view and the bonding process and the totally different approach, that it is good that the mother and the partner are there consciously experiencing that. Therefore, I think from this point of view general anesthesia has no place. Not so much because of a medical reason, but for psychological reasons I would not recommend general anesthesia.”

Fetal hemodynamic instability emerged as another concern due to suboptimal autoregulation, particularly in premature fetuses [51]. Anesthetics and opiates can trigger significant fluctuations in blood pressure and heart rate. Additionally, general anesthesia entails maternal risks such as intubation and ventilation complications, aspiration pneumonia, and higher blood loss [52].

Despite its use in APAW animal experiments, the duration and extent of fetal respiration suppression under general anesthesia for human transfers remain uncertain, according to participants (P39, P41). While epidural analgesia is not standard for vaginal premature deliveries, interviewees (P21, P22) highlighted its potential utility in alleviating device-related pain [53]. Considering all data and expert insights, a proposed approach (P36) suggests starting with general anesthesia for initial clinical transfers, potentially transitioning to spinal anesthesia as experience grows. To prevent ductus arteriosus closure, participants (P9–P16) recommended prostaglandin administration.

### Uterine relaxants

During the cannulation of the umbilical vessels, the blood supply from the native placenta to the fetus should be preserved as steady as possible. Therefore, preventing immediate placental detachment is critical. Reduced uterine contractions also aid in easier wound retractor insertion and fetal transfer into the bag. Expert interviews explored uterine relaxation solutions. While placental detachment was not expected in a short timeframe, optimizing all aspects during transfer was deemed important by one participant (P36). The mentioned medications for uterine relaxation included sevoflurane, nitroglycerin, pethidine, and ritodrine (P17, P18, P36, P37). Although uterine relaxation medication might be used, postpartum hemorrhage was not regarded as a large risk, and therefore interviewees (P36–P42) generally discouraged maternal arterial line use, except for rare conditions such as coagulation disorders (P37, P39, P40).

### Maternal blood loss

General anesthesia and/or uterine relaxants may lead to increased blood loss [49, 52]. To mitigate maternal blood loss, post-procedure uterine contraction is essential. Medications such as oxytocin, methylergometrine, sulprostone, and other prostaglandins can aid (P36–P38). Participant input (P38) also noted non-pharmacological interventions such as uterine massage, B-lynch, and uterine balloon tamponade as considerations.

### Rupture of the membranes

Preterm births stem from various causes; fetal membranes may rupture prematurely PPROM prior to surgery, exposing the fetus to infection risk [54]. Exploring whether a brief absence of amniotic fluid alone could trigger fetal-neonatal physiological transition requires further investigation. Thus, eligible patient criteria might need refinement. In vaginal delivery transfers, membrane rupture can be induced shortly prior to the procedure via an amniotomy hook [55]. An option is rupturing membranes just before device insertion, sealing around the cervix. When cervical dilation reaches approximately 6 cm or the fetal head diameter permits cervix passage (P9, P12), the liquid environment in which the infant is brought must be ready and near/attached to the mother.

### Fetal environmental exposure

While it is intended that during the transfer the fetus stays fully immersed in liquid, there is a chance of the fetal body not being completely or continuously submerged in AAF. Instances include umbilical cord cannulation and brief head exposure during uterine incision to transferbag attachment. Experts noted that these brief exposures, lasting seconds, are generally acceptable. However, attention must be given to premature infants’ rapid cooling (seconds to half a minute) triggering respiration and heightened infection risk upon skin contact with the exterior (P39–P42) [56, 57]. The fragility of the skin heightens the risk of skin tears (P42) and infections (P38–P40) [58].

### AAF flow into the uterus

During interviews and simulations, concerns were raised about AAF flowing from the pre-filled transferbag into the uterus, especially during cesarean sections, a concern confirmed by a liquid-based simulation. However, interviewees (P36–P42) generally view AAF entering the uterus as not problematic as it is a sterile fluid, and the uterus will not be filled under pressure. Mimicking the native amniotic fluid composition of water and electrolytes [59], complications such as transurethral resection syndrome [60] are not anticipated. After disconnecting the transferbag, AAF can be suctioned from the uterus, with uterine contractions aiding remaining fluid expulsion (P36, P37). One participant (P37) mentioned “I do not think the uterus will be three times its size because of this transfer. When you have delivered the baby and disconnected the transferbag, you […] only have to make sure to suction the amniotic fluid. After the procedure it will flow away [through the cervix]. Therefore, I do not think it will be a big problem”.

### Maternal positioning

Theoretically, a standard CS maternal position involves a 15° left lateral tilt to avoid vena cava and aortic compression [61, 62], averting hypotension. However, a 15° tilt is seldom achieved [63] (P40). The CS transfer procedure demands optimal maternal positioning for a near-horizontal pathway from the uterus to the transferbag. When the bag is upright and filled with AAF, fluid directly flows into the uterus. Proposals of a 30° left lateral tilt met reservations due to maternal instability and surgical access challenges, although some deemed it feasible under general anesthesia and proper practice (P41). Another suggestion proposed (P40) was to perform a temporary side turn at the moment that everything for the transfer of the perinate into the transferbag would be in place, i.e., just for a couple of minutes. Liquid-based simulations (phase V) verified that left lateral tilt and subsequent side tilting enabled a horizontal transferbag position, preventing excessive AAF influx to the uterus.

### Monitoring fetal vital functions

In a standard CS, the incision-to-delivery time is roughly 5 min [64]. Fetal vital functions are usually not monitored during this phase. Considering the longer duration and increased risks of the transfer procedure, fetal vital monitoring might be appropriate. Perinatologists interviewed (P36–P38) did not consider monitoring of vital functions necessary during the transfer. However, it was mentioned to be interesting from a scientific point of view, to be able to predict the prognostics more accurately. Anesthesiologists (P40, P41) stressed monitoring fetal heart rate, oxygen saturation, and blood pressure. Nevertheless, priority was given to a smooth, swift transfer due to the potential invasiveness and time consumption of monitoring methods.

Neonatologists (P39, P42) expressed interest in heart rate information during the transfer, while blood pressure and saturation were considered optional. Heart rate could serve as a threshold for initiating a rescue procedure in cases of prolonged bradycardia. Monitoring fetal heart rate aids decisions on continuing, pausing, or halting the transfer.

Participants suggested various monitoring methods: umbilical cord ultrasound Doppler (P39, P41), continuous abdominal ultrasound during CS (P37), abdominal ultrasound through the transferbag (P40, P42), and continuous cardiotocography during vaginal birth transfer (P6, P9–P16). Non-invasive cerebral hemodynamics via TD-NIRS was noted as promising for monitoring when the infant is in the transferbag [65].

### Hygiene

Prior APAW studies were hampered by sepsis development, partly due to fluid contamination [3]. For standard CS delivery, a sterile environment is crucial: operating under filtered air ventilation, with protective measures such as gloves and cover-ups. Interviewees believed this setup would preserve hygiene during transfers. While neonatologists typically are not in sterile outfits, their increased role, particularly in cannulation, suggests placing one sterile surgical attire post-transfer to minimize contamination (P39, P42). For vaginal delivery, extra hygiene measures are needed due to AAF contact with the vaginal microbiome. While not all studies support it [66, 67], intrapartum intravaginal lavage could reduce infant infection risk from maternal pathogens [68]. Some participants cautioned against vaginal lavage due to premature infant skin sensitivity (P21, P22), preferring non-iodine solutions (P21, P22). A physical barrier between the birth canal and perinate, coated with sterile agents, was also suggested (P17). During transfer, maternal amniotic fluid mixing with AAF might necessitate circulating AAF flow in the transferbag. Current APAW systems refresh AAF continuously to reduce microbial accumulation [5]. Participants recommended administration of antibiotics to further lower infection risk (P9, P12, P16, P39, P42).

### Cannulation and establishment of circulation

Umbilical cord length averages about 40 centimeters at 24 weeks of gestation [69]. Hence, if umbilical cord cannulation is performed, it must be done near the mother due to cord length constraints. Simulations (phases III and V) highlighted the need for the infant to stay close to the incision site post-delivery. Preserving adequate native cord length has been shown to be beneficial for improved cannula stability [10]. The duration of the cannulation was also mentioned (P42): “Safety requirements must be added [to the protocol]; how long can it take? If the cannulation lasts longer than X seconds, you must decide to convert to standard care”. In addition to performing the cannulation after the infant has been transferred into a transferbag, there is also the possibility of cannulation while the infant is still intrauterine (resembling the ex-utero intrapartum treatment) [70].

### Distribution of tasks and positioning of clinicians

Simulations (phases III and V) informed efficient task allocation and participant positioning around the operating table. Initial simulations involved two participants on each side (phase III). Simulations revealed tacit knowledge and guided discussions on dexterity, optimal sight, workload division, and transferbag handling, resulting in iterative refinements. During the simulations, it appeared the most optimal to have four persons involved in the transfer: a perinatologist and surgical assistant on either side of the operating table. The surgical assistants supported the perinatologists on corresponding sides. The perinatologist on the left side made the incisions (due to the left lateral tilt), and delivered the perinate in the transferbag, while the perinatologist on the right side inserted the retractors from the transfer tool. At the time of delivery, the staff on the right turned the mother on her side. Post-procedure, the right and left sides were split up into two teams. With the left side being responsible for the perinate and communicating with neonatologists, aid in the umbilical cord cannulation, and the right side oversaw maternal after care, delivered the placenta, and sutured the incisions (see Table 1).

**Table 1:** Proposed team for a CS transfer procedure and their tasks. Text in bold highlights the positions that have been simulated

Team		Function	Tasks
	Transfer to bag	Anesthesiologist	Provide anesthesia and analgesia Monitoring of the maternal vital functions
		Anesthesiology assistant	Assist the anesthesiologist
		**Perinatologist 1 (left)**	Perform the incisions Child delivery Communication with neonatologist
		**Surgical assistant 1 (left)**	Hand over components of transfer device Assist perinatologist 1
		**Perinatologist 2 (right)**	Inserting wound retractors Assist with the child delivery Sutures Monitoring blood loss
		**Surgical assistant 2 (right)**	Hand over the surgical instruments Assist perinatologist 2
		Sonographer	Monitoring fetal heart rate via ultrasound
Transfer to APAW system	Cannulation	Neonatologist	Monitoring vital functions of the fetus Cannulation of the umbilical cord
		Neonatology resident	Assist the neonatologist
		CIN nurse	Assist the neonatologist Involvement with parents
		Medical engineer	Technical support APAW system

### Checkpoints

To enhance communication and facilitate decision support, specific timed checkpoints were discussed in expert interviews (phase IV) and simulations (phase V). These included (1) instrument check, (2) instrument readiness and fetal update, (3) maternal and fetal status post-transfer, (4) fetal update post-cannulation, and (5) update post-established oxygenator circuit. The first checkpoint is thought to be of importance because of the additional, novel, equipment which might need more explanation and attention (P37). Additionally, the second checkpoint is at a crucial stage, “At the moment the child is in the transferbag, you have to look really carefully; is the situation of the child still good?” (P39). The main clinical staff member must inform everyone when he or she will start the delivery. The latter three checkpoints facilitate communication and decisions on continuing APAW treatment based on the maternal/fetal state. A specific time limit for cannulation was also considered (P42).

### Rescue procedure

Participants confirmed that cannulation entry would serve as the vascular access for medication administration during transfer, given time constraints and lack of alternative access (P36–P42). In case of failed cannulation or negative indications, the rescue procedure, involving stopping the transfer and switching to standard neonatal care, was recommended (P17). Emergency medications, including adrenalin, atropine, dopamine, sodium bicarbonate, and IV sodium chloride infusion, should be readily available (P36, P40).

### Transfer protocol

The protocols (Tables 2, 3, 4) and proposed setup are informed by the interviews, simulations, and literature study. Preliminary versions were refined through discussions and simulations before final testing (phase V, Fig. 2). Given the preclinical stage of APAW research, continued evolution of the protocols is necessary and serves as guidelines for procedure development.

**Table 2:** An iteration of the simulation protocol for a transfer by vaginal birth

Preparations	
1	Sign-in: Introduction of the entire team, patient, and procedure verification, list of allergies, and anticoagulation.
2	Positioning of the fetus and placenta is determined
3	Instrument check with an extra checklist for and familiarization of the materials.
4	Standard fetal and maternal monitoring modalities are employed such as heart rate and blood pressure monitoring as well as cardiotocography for maternal contraction and fetal monitoring before the procedure.
5	Ultrasound is performed on the Obstetric High Care (OHC) ward before the procedure is started to determine the position of the fetus.
6	The birth canal is measured for depth and width in the supine position to determine the suitability of the device. If measurements are within the designated range the procedure can be continued. If measurements are outside of this range the transfer procedure will be abandoned and a caesarian transfer or rescue procedure needs to be considered by the perinatologist.
7	The mother receives epidural anesthesia for pain management during the procedure.
8	Wait for the necessary cervical dilation by an estimate of the perinatologist. This is determined based on the gestational age of the fetus and its head circumference, ± 5–7 cm for a 24-week GA.
9	Cervical dilation is checked regularly.
10	When the necessary dilation is achieved the perinatologist informs operating theatre personnel to start preparations for the procedure.
11	Other members of the procedural team are also alerted to make their way to the operating theatre.
12	The patient is transported to the operating theatre.
13	All transfer-related necessary materials and devices are prepared.
14	The perineum and vulva are cleaned with sterile water.
15	The transfer device(s) is inserted into the birth canal.
16	Cervical dilation is checked for the final time.
17	Around this point, unruptured membranes are ruptured with the use of an amniotomy hook.
18	Ultrasound is performed to confirm the correct positioning of the device in relation to the spina iliaca of the pelvis [71].
Transfer	
19	Sufficient dilation and positioning of the device(s) are re-confirmed by the perinatologist
20	A sealed passageway in the birth canal is created for the transfer from the perinate from the natural uterus to the artificial liquid environment (transferbag from this point onward).
21	The perinatologist begins filling of the transferbag with AAF to keep the head of the perinate submerged.
22	Contractions, maternal pushing, and guidance by the perinatologist’s hand(s) will ensure the perinate to slide into the birth canal and subsequently into the transferbag.
23	The perinate will now be fully encapsulated by the transferbag.
24	The transferbag is removed from the birth canal. Umbilical cord is still connected to the placenta and fetus.
25	The transferbag is placed in a stable position to allow for safe cannulation. In the meantime, temperature change of the perinate (and AAF) should be prevented.
Cannulation and afterbirth	
26	Cannulation of all three umbilical cord vessels should take place within a few minutes to avoid asphyxia [72].
27	The perinatologist proceeds to guide the delivery of the placenta and suture possible ruptures.
28	The perinate is transferred to the LFC of the APAW system where monitoring, oxygenation, and nourishment of the perinate are fully taken over.

**Table 3:** An iteration of the simulation protocol for a transfer by cesarean section delivery

Preparations		
1	Sign-in: Introduction of the entire team, patient, and procedure verification, list of allergies, and anticoagulation.	
2	Positioning of the fetus and placenta is determined	
3	Instrument check with an extra checklist for and familiarization of the materials.	
4	Standard fetal and maternal monitoring modalities are employed such as heart rate and blood pressure monitoring as well as cardiotocography for maternal contraction and fetal monitoring before the procedure.	
5	Inform operating personnel to start preparations for the procedure.	
6	Other members of the procedural team are also alerted to make their way to the operating theatre.	
7	The patient is then transported to the operating theatre.	
8	Patient is placed in a 15° left lateral tilt position.	
9	Preoxygenation of the patient.	
10	The anesthesiologist administers general anesthesia. • Propofol induction • Sevoflurane maintenance • Opiates • Intubation	
11	Administer prophylactic antibiotics	
12	Placing body support components (shoulder, hip, gel pads between feet and arms)	
13	Inserting catheter	
14	The operating field is prepared according to standard protocols (NVDV, Dutch Association for Dermatology and Veneorology)	
15	Prepare all the necessary CS transfer materials.	
16	Start ultrasound monitoring	
	*Communication moment 1 (transfer team)—start operation*	
Transfer		
17	The surgeon makes a Pfannenstiel incision through to the peritoneum according to standard protocol (Dutch Pediatrics Association), splitting the abdominal muscles (until the abdominal cavity)	
18	Blood is tamponed from the incision site to avoid cloudiness of the (artificial) amniotic fluid.	
	*Communication moment 2 (transfer team)—uterus incision*	
19	The incision in the uterus is made. The width of the incision is based on the diameter of the fetal skull (P29).	
20	Amnioinfusion into the native uterus to keep the fetal head submerged.	
21	The transferbag is filled with AAF before the perinate is transferred.	
22	Increase oxygen percentage (fetal preoxygenation)	
23	Mother is manually tilted to her left side.	
24	The perinate is delivered while the breathing reflex is prevented and other environmental stimuli are shielded as much as possible.	
25	In case of severe uterine contraction, intravenous nitroglycerin can be given at this point for uterine relaxation to facilitate fetal extraction [73]. Dosage is at the discretion of the anesthesiologist	
26	The infant is taken from the natural uterus completely into the transferbag.	
27	The transferbag is closed from exterior exposure to avoid AAF from leaking or exterior factors to enter.	
28	The transferbag is placed in a stable position to allow for cannulation before the perinate is placed in the more permanent APAW system. In the meantime, temperature change of the perinate (and AAF) should be prevented.	
29	Mother placed in the supine position.	
Cannulation, installation, and suturing		
30	Ultrasound monitoring of heart rate via umbilical cord	
31	Neonatologist moves to the operation table.	
	*Communication moment 3—decision to proceed with APAW treatment*	
32	Splitting into two teams: perinatal team and maternal team	
Perinatal team	Maternal team	
A	Preparing the umbilical cord	A Suction AAF from uterus
B	Cannulation of the umbilical cord	B Wait for placental delivery until cannulation succeeded
C	Clamping of the umbilical cord	C Administer oxytocin
*Communication moment 4 (technical support for adjustments to APAW system)*		D Uterus massage
D	Administering of medication when necessary	E Placental delivery through controlled cord traction
E	Bring perinate to APAW system	F Blood loss monitoring, additional medication is given if necessary
		G Suturing of the uterus and skin
Wrap up		
33	Stop anesthesia, extubate the mother	
34	Sign-out: count materials, after-care policy	
35	Evaluation of procedure and feedback

**Table 4:** Protocol for a rescue procedure after starting the transfer procedure

1	As soon as the perinate is exposed to air/as soon as the umbilical cord is cut, a timer is set.
2	The (now) newborn is placed onto the workstation and immediately wrapped in an isolation bag and fitted with a cap. The isolation bag will only be removed from the incubator once the newborn has arrived at the neonatal intensive care unit.
3	Breathing and circulation are observed. Necessary interventions are done according to “Airway, Breathing, Circulation” and neonatal resuscitation protocols [74] (NVK and NRR).
4	A hole in the isolation bag allows the hand of the newborn to be pulled through so that it can receive an intravenous drip.
5	Pulse/oximeter is attached to the right hand/arm of the newborn for measurement of preductal values. The device is only turned on after it has been attached to the newborn.
6	The newborn is placed into the transport incubator.
7	Parents are shortly informed of the situation of the newborn and if its condition allows it, parents can briefly see their child.
8	The newborn is brought to the neonatal intensive care unit.

Our proposal divides CS and vaginal transfer protocols into stages with three dedicated teams (Table 1) for optimized workflow. These phases would include (1) preparation, (2) transfer, and (3) cannulation and placement in the APAW system. Within the current study, only the preparation, transfer phases, and the involvement of the first team were simulated. The first team monitors the mother and fetus. The second team handles cord cannulation and perinate stabilization. The third team places the perinate in the APAW system.

The estimated personnel for a full CS transfer procedure are eleven medical staff members with specific expertise (see Table 1). As this would be logistically and economically challenging, further research is required to understand the feasibility and determine whether this is an ideal setup in practice. Similarly, for a transfer procedure after vaginal birth, the same teams are needed, yet the number of team members may be reduced to eight staff members: two perinatologists, an obstetrics nurse, an anesthesiologist with an assistant, a neonatologist with an assistant, and a technical physician.

We propose to enable two transfer procedures depending on the delivery type: vaginal (see Table 2) and CS (see Table 3). Although vaginal birth is the dominant procedure for the delivery of premature infants delivery globally [75], several considerations led us to develop a protocol for two delivery modes. A CS delivery cannot always be replaced by a vaginal delivery and vice versa. Excluding vaginal delivery in advance limits the application and could force many women to have a CS that otherwise would not have occurred [4]. Furthermore, in current practice, CS can be chosen for various reasons, including electives, making it unjustified to enable only vaginal delivery. Further research must show whether a vaginal delivery is indeed compatible, given that the fetal-to-neonatal transition could already be initiated at labor onset by exposure to (hormonal and mechanical) factors associated with parturition [76].

The discontinuation of transfer to an APAW system necessitates a switch to conventional neonatal care (Table 4) due to human/technological errors or medical necessity. The rescue protocol aligns with established guidelines for extremely premature infants (Máxima Medical Center, the Netherlands) (NVK, Dutch Pediatric Association) (NRR, Dutch Resuscitation Council) [74].

To enhance team communication and comprehension, we propose incorporating scheduled, brief breaks during the procedure. Prior research underscores their contribution to treatment quality and patient safety [77], a concept that participants embraced.

### A framework for shaping future care

**Fig. 4:**
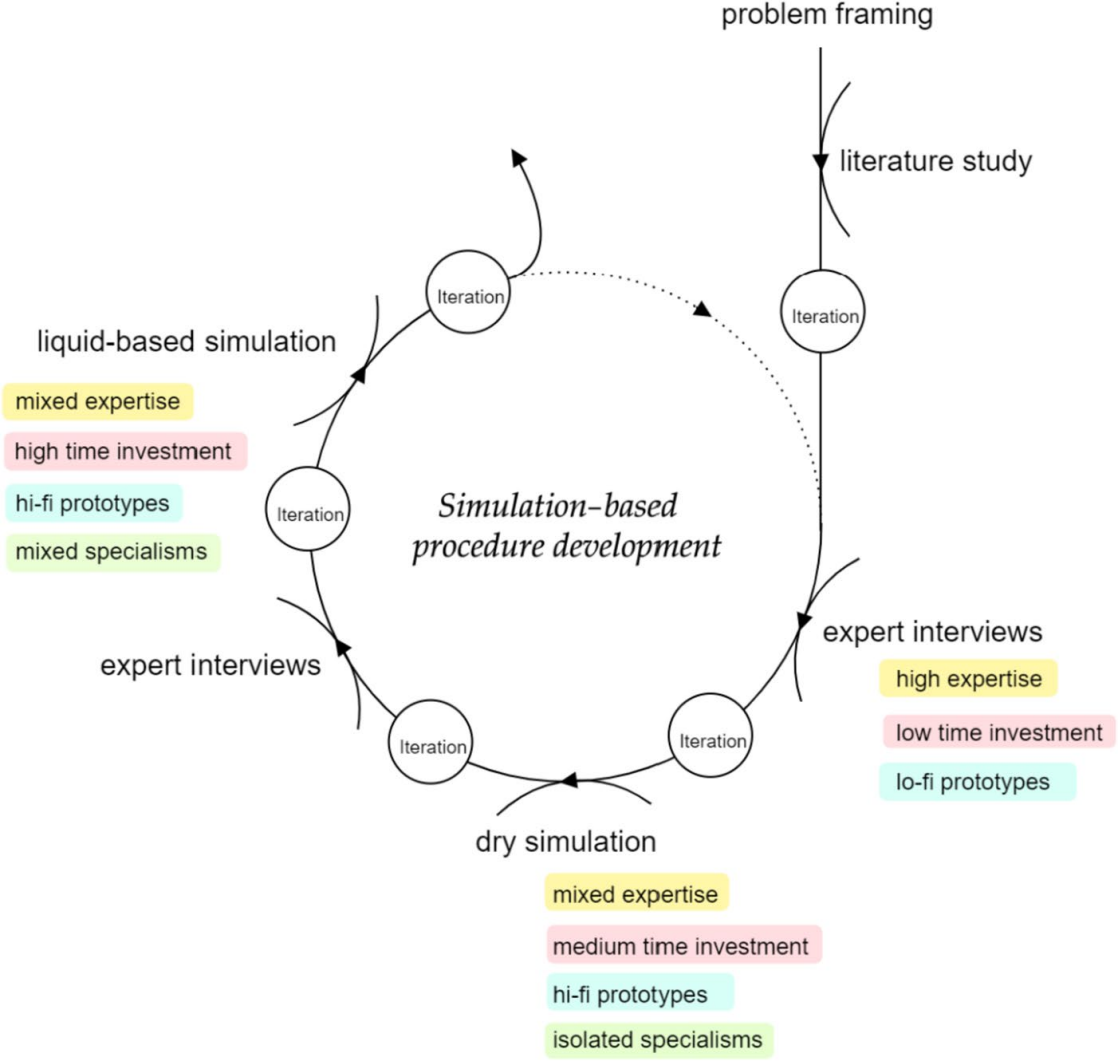
Simulation-based procedure development cycle, with intermittent literature study, interview, and simulation cycles. Legend: Hi-fi and lo-fi refer to high and low fidelity, respectively

Figure 4 outlines a simulation-based co-creation approach involving diverse stakeholders, aimed at iterative knowledge development, adaptable to various clinical interventions beyond APAW technology. Simulation-based development requires mediation skills to address stakeholder priorities, particularly during initial technology phases. Isolated single-actor simulations focus on specialty-specific elements while multi-actor sessions with composite teams facilitate interdisciplinary collaboration, communication, testing of cross-discipline tasks [78] and promote consensus building. Minimizing interruptions to clinical practice and adjusting time investment as needed is crucial. Participant expertise levels evolve through iterations. Co-creation sessions with medical residents can give enough feedback to start concept development, whereas for specific aspects senior clinical professionals are needed to provide targeted input. With this simulation-based development framework, both technical and communication (teamwork) domains were addressed and may better equip a medical team with both soft and hard skills.

## Discussion

This study presents the first description and simulation protocol of an APAW transfer procedure and employs simulation-based participatory methods for its development. We hypothesized that the use of medical simulation will elicit realistic user insights regarding a transfer procedure and improve the development of a protocol and guidelines for obstetric tools. Clinicians actively participated in the design process to create a step-by-step procedure that matches their workflow and is suited to the needs from a clinician’s perspective. This initial protocol is a foundation, and future research stages should involve patient representatives in expert interviews and simulations to incorporate their insights.

Simulation-based development of novel technologies has long been conducted in the field of spaceflight, as early as during NASA’s Apollo project with simulation facilities, to test functionality, reliability, and usability [79]. Within healthcare, studies have shown the effective use of simulation-based user-centered design for the development of hospital planning to improve patient safety [21, 22], and in the development of medical devices for pediatric intubation [23]. While these examples demonstrate the success of participatory simulation, these applications have not yet included the development of unprecedented extra-corporeal life support technologies. Our work shows that participatory design is an insightful approach to the development of APAW, where user engagement can lead to a comprehensive understanding of the challenges, opportunities, and risks. The method allows stakeholders to engage with complex systems in a safe and controlled environment. Participants may take on different roles and interact with one another to understand how the system works, identify potential problems or opportunities, and develop solutions.

By immersing researchers and clinicians in the operating theater environment, we aimed to gather realistic user insights, hence reducing the need for future protocol modifications when applying a protocol in practice. By gaining direct input from user perspectives, this study identified twelve themes as needing intervention from the existing obstetrics protocol for extremely preterm birth. These include the management of anesthesia, uterine relaxation, maternal blood loss, fetal environmental exposure, maternal positioning, AAF flow into the uterus, monitoring of fetal vital functions, hygiene, mode of transfer, cannulation, rupture of membranes, and the rescue procedure. During the simulations, participants were better able to imagine potential risks, suggest additional protocol requirements, and envision tangible improvement points compared to sessions held in an out-of-context interview setting.

A key insight is to prevent unnecessary administration of medication and the uncertain duration of the suppression by designing the transfer procedure in such a way as to physically prevent air exposure to the human infant. Direct delivery into a transferbag filled with AAF, creating a tunnel, achieves this.

This study also refined the required team composition from preparation to placing the infant in the APAW system. Logistical coordination can be challenging because of the hectic schedules of the many medical professionals that need to be engaged to form a comprehensive overview of the user’s needs. Portable simulation setups minimize disruption to clinical practice, reduce time investment, avoid unnecessary occupation of operating theatres, and engage multiple clinicians at once. Using simulation is also cost and time-effective while preparing and conducting validation studies. Sessions can be performed on a single simulator several times; it does not need to be planned months ahead, nor does it require all medical experts to be present at the same time. Effective task division could be analyzed through research on mental workload, such as through the NASA task load index [80] or its surgical equivalent SURG TLX [81]. Participants can be videotaped from multiple angles, which allows for post-simulation analysis of human factors such as effective task division.

Placing an infant into an extra-corporeal life-support system is a high-risk procedure. Before a first patient could be treated, procedure development requires the understanding of both high and low risks and incidence scenarios. Due to ethical, clinical, practical, and economic considerations, animal studies and/or conventional clinical trials for APAW development are far from straightforward. A simulated environment has many degrees of freedom and may provide an additional research platform. This allows the ability to practice with complex scenarios, such as (simulated) technical failures of the equipment, certain patient pathologies, or other complex clinical situations.

Most of the attention in APAW development and debate has been drawn to the long-term implications, while short-term ethical concerns, such as animal well-being, should not be overlooked. In addition, findings from animal studies do not guarantee consistent findings in human investigations. Longitudinal human clinical trials would still be necessary upon clinical implementation [12]. Therefore, simulation-based development could minimize the number of animal studies needed, bridge the gap between animals and human trials, and thus contribute to a reduced risk for the first human participants.

### Rehearsing future care

Although the development of APAW technology is still in its infancy, the developed simulation technology for a potential treatment is not limited to procedure and system development and can already be used for medical training. During medical simulation training a broad range of medical scenarios can be initiated and assessed [82]. Simulation could allow for practicing and fine-tuning task handling. Another advantage of the use of simulators is the possibility of measuring the learning curve and performance of the user through longitudinal data acquisition during the different design iterations. This could be done by quantifying the proficiency of a certain task as a function of time-to-completion, and the parameter values of vital organs can be monitored to visualize the reaction-time of a certain task to presented symptoms [16, 83]. Physical fetal manikins with embedded digital twin technology [84] can, in this context, be used to train clinical decision-making. Furthermore, team dynamics can be rehearsed for alignment.

### Limitations

Translating the medical environment to a simulation involves substituting patients, protocols, tools, and pathologies with manikins, simulation protocols, simulation tools, and scenarios. This translation step will inevitably lead to a decrease in fidelity since an exact artificial replica with all possible functions of the human body cannot be replicated. Maintaining a suspension of disbelief during a simulation session is therefore important to allow participants to focus on the learning objectives rather than being distracted by the limitations of the simulation equipment [85]. Verification of the realism of the simulation is challenging, as data acquisition during pregnancy is generally limited by non-invasive measurements to protect the mother and fetus. To mitigate this challenge, thorough literature research, continuous feedback (on simulation realism) from clinicians, and the collection of clinical information and data is crucial. Data-driven manikins can also enhance fidelity for early-phase APAW development.

Future simulation research should allow cannulating the umbilical vessels, which was not part of the current study. APAW studies have described the cannulation in different settings, both whilst the animal is still in-utero or ex-utero. Although the simulation sessions of this study only enacted ex-utero cannulation, this should be extended to include in-utero cannulation as well. The majority of animal studies demonstrating successful and rapid cannulation of the umbilical vessels to the extracorporeal circuit have been conducted on sheep, whose umbilical anatomy differs from that of humans (four vessels instead of three), with piglet models being more similar to humans. Simulation technology can be complementary to this research. Through the advancement of the fidelity of the umbilical cord phantoms, such as improving its biomechanical properties, more knowledge can be yielded on cannula design, procedures, and skills.

The simulation protocol created in this study is a dynamic starting point for validation and training.

It serves as a discussion piece and will guide further development of the transfer procedure. Clinical testing is required to ascertain the effectiveness of the procedures in maintaining fetal physiology and preventing fetal-to-neonatal transition, particularly in reducing tactile, thermal, hormonal, and environmental stimuli triggering breathing initiation.

The researchers took the primary roles as simulation designers and used semi-structured interviews and simulation guidelines to foster open discussion and exploration of novel approaches and insights. Due to the semi-structured set-up, bias may have occurred while moderating to engage further in certain topics.

### Future outlook

The integration of physical manikins with digital twins, incorporating computational models of fetal physiology, could offer a larger array of scenarios (e.g., pathologies) and thereby improve potential for treatment development and training. This could further enrich the simulation-based development method with data-based evaluation of usability, effectiveness, safety, hygiene, and comfort; identifying hazards and human errors, establishing task division among professionals, and ultimately improving the design and treatment protocol. By using real-time feedback from the hybrid manikins regarding participants’ actions and performance, the skill of immediately anticipating new situations can be trained. This method can also be applied to other technical developments in medicine.

Whereas this study engaged primarily with stakeholders from clinical practice, future studies should also include the involvement of patient organizations during the simulations. Through a value-sensitive design approach, the technology and procedure can be better aligned with human patient values [4, 12].

## Conclusions

Transferring an infant from the mother’s womb to a liquid-based perinatal life support system is an unprecedented task and a, very likely, high-risk procedure for which no protocol exists. Most current APAW research is being reviewed in an animal laboratory setting, which might not reflect human patient care. Medical simulation could be used to approximate the human condition and provide a safe and controlled environment to form a shared understanding of procedure requirements. This study demonstrates a simulation-based user-centered development method for designers to co-create with clinicians in early-phase medical treatment development. The presented research focused on protocol development for a transfer procedure to an APAW system and provided a framework that could serve as a basis for procedure development in general. These simulation protocols may also form a basis for the development of medical devices. In summary, this approach fostered collaborative discussions on a variety of themes, including management of anesthesia, hygiene, maternal and fetal safety, teamwork, and effectiveness of the transfer.

### Supplementary Information

**Additional file 1.** Questions asked in expert interviews phase IV.

**Additional file 2.** A statement of ethics approval.

## Data Availability

Questions asked in expert interviews phase IV can be found in Additional file 1.pdf Other datasets used and/or analyzed during the current study are available from the corresponding author upon reasonable request.

## Abbreviations

AAF: Artificial amniotic fluid
APAW: Artificial placenta and artificial womb
AP: Artificial placenta
AW: Artificial womb
CS: Cesarean section
P1-P49: Participant 1–participant 49
VB: Vaginal Birth
