# Supplementary material for: Simulation-based development: shaping clinical procedures for extra-uterine life support technology
Source: Adv Simul (Lond). 2023 Dec 2;8:29. doi: 10.1186/s41077-023-00267-y (PMC10693037; doi:10.1186/s41077-023-00267-y)
Supplement: Supplementary file 1 — Additional file 1. Questions asked in expert interviews phase IV. [file 41077_2023_267_MOESM1_ESM.docx]

Additional file 1. Questions asked in expert interviews phase IV.

| 1 | Can maternal general anesthesia suppress the fetal respiratory stimulus? (Despite short-term exposure to ambient air and mechanical stimulation or other triggers) |
| --- | --- |
| 2 | What considerations do you make in choosing or not choosing general anesthesia and what would this mean for the mother and fetus? |
| 3 | In addition to respiratory consequences, do you foresee any other risks to short-term fetal environmental exposure during transfer? |
| 4 | Is a maternal position of 30 degrees left lateral tilt feasible? |
| 5 | Is it a risk if artificial amniotic fluid enters the maternal uterus and the maternal  abdominal cavity during transfer? (Risks of TURP syndrome, disruption in ions, stretching of the uterus). |
| 6 | Is it necessary to insert an arterial line in the mother? |
| 7 | Is it necessary to monitor the vital sings of the fetus during the transfer? (What parameters would be insightful?) |
| 8 | Is it necessary to create vascular access during or prior to cannulation to administer medication to the fetus? If so, which medication and why? |
| 9 | What feedback do you have on the current transfer protocol? |
| 10 | What is your opinion regarding the stop/pause moments? |
| 11 | Are there any additional hygiene measures (relative to normal CS) that should be observed? |
