# Supplementary material for: Simulation-based development: shaping clinical procedures for extra-uterine life support technology
Source: Adv Simul (Lond). 2023 Dec 2;8:29. doi: 10.1186/s41077-023-00267-y (PMC10693037; doi:10.1186/s41077-023-00267-y)
Supplement: Supplementary file 2 — Additional file 2. A statement of ethics approval. [file 41077_2023_267_MOESM2_ESM.pdf]

máxima mc

medisch ethische toetsingscommissie
secretariaat
telefoonnummer: 040888 9528
e-mail:metc@mmc.nl

Mevrouw Dr. Ir. M.B. van der Hout
Obstetrie & Gynaecologie
Máxima MC
Locatie Veldhoven

Datum:	30 september 2020
Brief nummer:	2020-184
Betreft:	WMO-plichtigheid
Studie:	Protocolontwikkeling Perinatal Life Support
METC nummer	N20.109

Wij verzoeken u om bij verdere correspondentie bovenstaand METC nummer te gebruiken.

Geachte mevrouw Van der Hout,

De medische ethische toetsingscommissie (METC) van Máxima MC heeft bovengenoemd onderzoeksvoorstel ontvangen op 29-09-2020. Het dagelijks bestuur van de commissie is tot de conclusie gekomen dat het onderzoek niet onder de werkingssfeer van de *Wet medisch wetenschappelijk onderzoek met mensen* (WMO) valt.

Volledigheidshalve benadrukt de METC dat de studie niet is beoordeeld op relevantie, kwaliteit en conformiteit met overige mogelijk van toepassing zijnde wet- en regelgeving (zoals de WGBO, de AVG en de code 'Goed Gebruik').

Alle wijzigingen in deze studie dienen opnieuw aan de METC te worden voorgelegd.

Volledigheidshalve maken wij u erop attent dat het onderzoek pas mag worden uitgevoerd nadat u schriftelijk toestemming heeft gekregen van de instelling.

Ik hoop u hiermee voldoende geïnformeerd te hebben.

Met vriendelijke groet,

Mevr. Y.I.C. (Yolanda) de Haan
Ambtelijk secretaris METC

To whom it may concern,
The Daily Board of the Medical Ethics Committee Máxima MC (hereafter the Committee), has reviewed the above mentioned research proposal. As a result of this review, the Committee informs you that the rules laid down in the Medical Research Involving Human Subjects Act (also known by its Dutch abbreviation WMO), do not apply to this research proposal.
